# Human immunodeficiency virus type 1 Vpu and cellular TASK proteins suppress transcription of unintegrated HIV-1 DNA

**DOI:** 10.1186/1743-422X-9-277

**Published:** 2012-11-19

**Authors:** Nkiruka Emeagwali, James EK Hildreth

**Affiliations:** 1 Center for AIDS Health Disparities Research, Meharry Medical College, Nashville, TN, 37208, USA; 2 Department of Molecular and Cellular Biology, College of Biological Sciences, University of California, Davis, One Shields Ave, Davis, CA, 95616, USA

**Keywords:** HIV-1, Viral protein U, Unintegrated HIV-1 DNA, HIV-1 transcription Twik-related Acid Sensitive K+ proteins

## Abstract

**Background:**

Unintegrated HIV-1 DNA serves as transcriptionally active templates in HIV-infected cells. Several host factors including NF-κβ enhance HIV-1 transcription. HIV-1 induced NF-κβ activation can be suppressed by viral protein U (Vpu). Interestingly HIV-1 Vpu shares amino acid homology with cellular Twik-related Acid Sensitive K+ (TASK) channel 1 and the proteins physically interact in cultured cells and AIDS lymphoid tissue. Furthermore, the first transmembrane domain of TASK-1 is functionally interchangeable with Vpu and like Vpu enhances HIV-1 release.

**Results:**

Here we further characterize the role of TASK channels and Vpu in HIV-1 replication. We demonstrate that both TASK channels and Vpu can preferentially inhibit transcription of unintegrated HIV-1 DNA. Interestingly, TASK-1 ion channel function is not required and suppression of HIV-1 transcription by TASK-1 and Vpu was reversed by overexpression of RelA (NF-κβ p65).

**Conclusion:**

TASK proteins and Vpu suppress transcription of unintegrated HIV-1 DNA through an NF-κβ-dependent mechanism. Taken together these findings support a possible physiological role for HIV-1 Vpu and TASK proteins as modulators of transcription of unintegrated HIV-1 DNA genomes.

## Background

Human immunodeficiency virus type 1 (HIV-1) replication involves a series of highly orchestrated steps that are regulated by both viral and cellular factors. Like other retroviruses, HIV-1’s genome is reverse transcribed before the proviral DNA integrates into host cell chromatin. HIV-1 proviral DNA preferentially integrates into open transcriptionally active sites in the host cell chromatin where robust viral gene transcription can occur. Integration of HIV-1 DNA, mediated by viral integrase, allows the viral genome to persist throughout the lifespan of the cell. However, despite the critical role of integration in the lifecycle of all retroviruses, during the course of HIV-1 infection the majority of viral DNA remains unintegrated [1].

Unintegrated HIV-1 DNA (uDNA) is generated during virus replication and serves as DNA templates for transcription. The majority of viral uDNA genomes remain linear. Linear HIV-1 DNA, the substrate for integration, is found in both the cytoplasm and nucleus and can be degraded or modified by host factors [1-3]. uDNA can circularize by homologous recombination [4] or ligation of interrupted reverse transcription intermediates [5] to form 1-LTR circles. uDNA can also circularize by non-homologous end joining DNA repair events to form 2-LTR circles [6,7]. In addition, formation of 1-LTR and 2-LTR circles can result from activity of host factors involved in DNA repair. Circular forms of the viral genome are found exclusively in the nucleus. Interestingly, the host restriction factor APOBEC3G has been shown to cause a 2-fold decrease in 2-LTR circle formation in cells infected with Δvif HIV-1 [8].

In HIV-1-infected patients, high levels of uDNA can be found in the blood, lymphoid tissues, and brain [1,6,9-12]. uDNA also accumulates in non-dividing cells such as macrophages and resting T cells but is lost in dividing cells through dilution during cell division [13-15]. Clinically, detection of high levels of uDNA in the brain is associated with development of AIDS dementia and correlates with a decline in CD4+ T cells. In addition, uDNA templates are increased in HIV-infected cells of patients given ART regimens with the integrase inhibitor, Raltegravir. The accumulation of uDNA in patients appears to be independent of viral load [16,17].

Several studies have characterized important roles for uDNA with respect to HIV-1 infection [18-24]. Linear uDNA is the form used by HIV-1 integrase to incorporate the viral genome into host cell chromatin. uDNA can contribute to virus genetic diversity by providing templates for viral genome complementation of the integrated HIV-1 genome during virus assembly and production [25,26]. Transcriptional activity in uDNA is lower than that of integrated genomes. However, early gene products of HIV-1 infection, Tat and Nef, are transcribed and translated in cells containing only uDNA [20,24,27,28]. Nef protein production from uDNA is sufficient to promote T cell activation, down regulate CD4 and enhance HIV-1 infection [24]. Tat, an important viral protein in HIV replication, is initially produced from uDNA. Tat production from uDNA transactivates the HIV-1 LTR of both uDNA and integrated HIV-1 DNA [29].

Transcription of uDNA is enhanced by viral protein R (Vpr) a late gene product of HIV-1 infection. Interestingly both virion-associated Vpr and newly synthesized Vpr preferentially increase transcription of nef from uDNA templates [30]. Another late gene product of HIV-1 infection is viral protein U (Vpu). Vpu promotes the efficient release of virus particles from the cell surface, induces the degradation of CD4 in the ER and suppresses the activation of NF-κβ [31-33]. Vpu also restricts the function of several host proteins including tetherin and Twik-related Acid Sensitive K+ channel-1 (TASK-1) [34,35]. TASK proteins are a part of the family of two-pore domain background potassium (K(2P)) channels.

TASK channels are widely expressed by many cell types and establish resting membrane potential and cell excitability. They are functional as dimers and have been shown to form homo- and heterodimers *in vivo*[36]. Hsu et al. demonstrated that HIV-1 Vpu shows a high degree of sequence similarity to the first transmembrane domain of TASK-1 (Ttm1) [34]. In addition, Vpu can self-assemble into homo-oligomeric complexes and form ion channels in lipid bilayers. Interestingly Vpu’s ion channel function is not required to antagonize CD317-mediated restriction of HIV release [37,38]. In their study characterizing the homology between Vpu and the first transmembrane domain of TASK-1, Hsu et al. demonstrated that Vpu and Ttm1 were each capable of functional inhibition of the other. Furthermore it was found that cells infected with Vpu-deleted HIV-1, NL4-3 Udel, produced low levels of virus and this was reversed when Ttm1 was expressed in *trans*[34].

These observations suggest that some form of molecular piracy may have occurred during the evolution of HIV-1 infection. Here we demonstrate for the first time that TASK proteins and Vpu preferentially suppress transcription of uDNA. Our results have important implications for further understanding host-pathogen interactions that regulate HIV-1 replication and pathogenesis.

## Results

### Overexpression of TASK-1 suppresses HIV-1 replication

**Figure 1:**
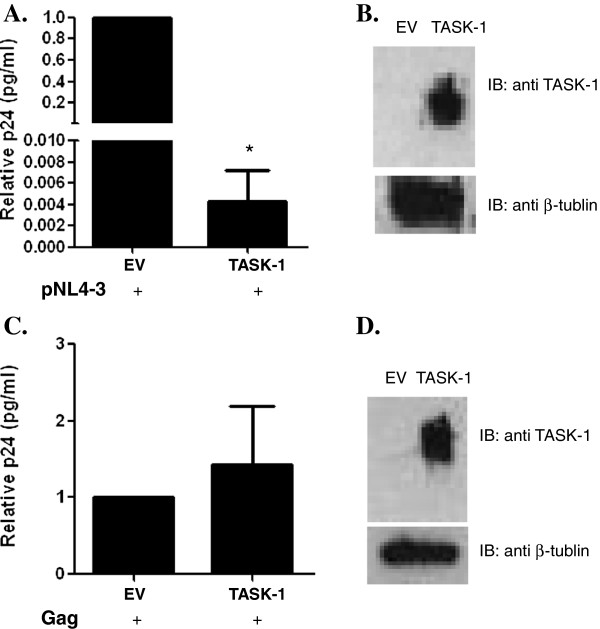
**TASK-1 suppresses HIV-1 replication. A**. 293T cells were co-transfected with pNL4-3 and TASK-1 expression vector or empty vector. After 48 hours supernatants were collected and a p24 ELISA was performed. Data are normalized to the empty vector control (EV). **B**. TASK-1 expression in transfected cells. 293T cells were harvested and lysed with buffer containing protease inhibitors. Equal amounts of protein from cell lysates were separated by 4-12% SDS-PAGE and transferred onto nitrocellulose membranes. TASK and β-tubulin proteins were detected by immunoblotting using polyclonal antibodies and visualization by chemilumenescence. **C**. TASK-1 does not decrease HIV-1 Gag virus-like particle release. 293T cells were co-transfected with plasmid encoding Gag driven by CMV promoter and TASK-1 or empty vector. After 48 hours supernatants were collected, and a p24 ELISA was performed. Data are normalized to the empty vector control (EV). **D**. TASK-1 expression in transfected cells from (C). 293T cells from (C.) were harvested and lysed with a detergent buffer containing protease inhibitors. Equal protein amounts of cell lysate were separated by 4-12% SDS-PAGE and transferred to nitrocellulose membranes. TASK and β-tubulin proteins were revealed by immunoblotting using polyclonal antibodies followed by chemilumenescence detection. (* P < .0001; Student T test was used to determine statistical significance).

We sought to extend this finding from a previous study that demonstrated that TASK-1 suppressed HIV replication [34]. 293T cells were cotransfected with HIV-1 pNL4-3 and TASK-1. After 48 hours, supernatants were collected and HIV-1 was quantified with a capture p24 antigen ELISA. Our results show that virus release was reduced by 82.5% in HIV-1-infected cells overexpressing TASK-1 protein compared to cells transfected with the empty vector control (Figure 1a, 1b). This result confirms the findings of Hsu and co-workers who demonstrated a decrease in HIV-1 release from HeLa cells co-transfected with pNL4-3 and TASK-1. We obtained similar data when 293T cells were co-transfected with pNL4-3 along with human TASK-3, murine TASK-1 or murine TASK-3. In addition, we also accessed the effect of TASK overexpression on cell viability and observed no decrease in viability resulting from TASK overexpression (data not shown). These results indicate that the effect of TASK-1 on HIV-1 release does not result from cellular toxicity and is shared with other TASK proteins, independent of species.

Assembly and budding of progeny virions from infected cells comprise the late stages of HIV-1 biogenesis and are multi-step events driven by the viral protein Gag. Indeed, transient expression of Gag alone is sufficient for the formation of virus-like particles (VLPs) consistent with the central role of Gag in particle assembly and release. To evaluate the effect of TASK proteins on the late stages of the HIV-1 life cycle, particle release from 293T cells co-expressing Gag driven by a CMV promoter and TASK-1 was evaluated. We observed no difference in Gag-mediated VLP release from cells in the presence or absence of TASK-1 protein (Figure 1c, 1d). Similarly using provirus HIV-1 SG3 containing a neomycin phosphotransferase (NPT) gene under the transcriptional regulation of SV40 we observed no change in NPT protein levels with TASK protein expression while HIV-1 Gag protein under the control of the LTR promoter was significantly decreased (data not shown). These findings indicate that the effect of TASK proteins on HIV-1 replication is not due to inhibition of Gag assembly and release. Furthermore TASK protein expression preferientally suppresses gene transcription driven by the HIV-LTR but not transcription from CMV or SV40 promoters.

Since the late stages of virus replication were not affected by TASK protein overexpression we examined the effect of TASK protein expression on the earlier stages of HIV-1 replication. 293T cells were cotransfected with pNL4-3 and TASK-3 or empty vector. After 48 hours intracellular Gag from cell lysates was quantified with a capture p24 antigen ELISA. We found that overexpression of TASK proteins decreased intracellular Gag by over 83% (data not shown).

### TASK-1 protein suppresses HIV-1 transcription

**Figure 2:**
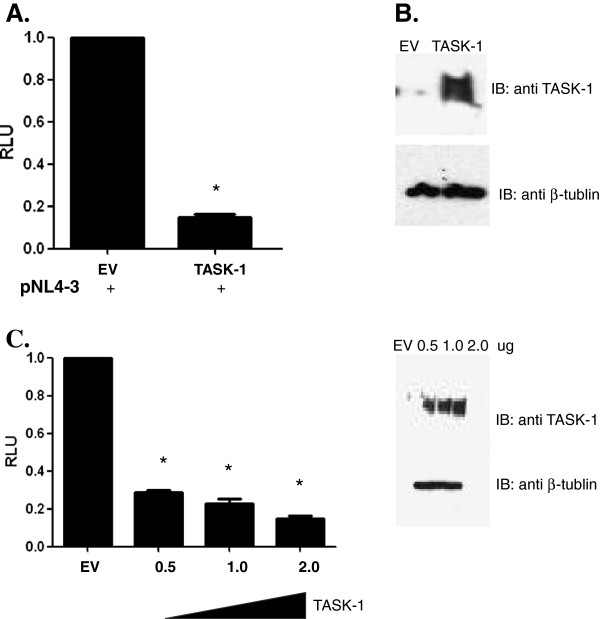
**TASK proteins suppress HIV-1 transcription.A**. 293T cells were transfected with pNL4-3Luc and TASK-1 expression vector or empty vector (EV). After 48 hours cells were lysed and a luciferase assay was performed to determine the effect of TASK-1 on HIV-1 transcription. Data are normalized to the empty vector control (EV). **B**. Transfected 293T cells were harvested and lysed with a detergent buffer containing protease inhibitors. Equal protein amounts of cell lysate were separated by 4-12% SDS PAGE and transferred to nitrocellulose membranes. TASK and β-tubulin proteins were revealed by immunoblotting with polyclonal antibodies followed by chemilumenescence detection. **C**. 293T cells were transfected with pNL4-3Luc and 0.5ug, 1ug, or 2ug TASK-1 or empty vector. After 48 hours cells were lysed and a luciferase assay was performed. Data are normalized to the empty vector control (EV). D. Transfected 293T cells from (C.) were harvested, lysed and equal amounts of protein from cell lysates were subjected to 4-12% SDS PAGE. After transfer to nitrocellulose membranes, TASK and β-tubulin proteins were revealed by immunoblotting with polyclonal antibodies followed by chemilumenescence detection. (* P < .0001; Student T test was used to determine statistical significance).

One possible explanation for the decrease in both extracellular and intracellular Gag expression in cells overexpressing TASK proteins is suppression of HIV-1 gene transcription. To test this possibility, a reporter virus construct derived from pNL4-3 in which the luciferase gene is substituted for nef (pNL4-3Luc), was cotransfected into 293T cells along with TASK-1. After 48 hours, the transfected cells were lysed and a luciferase assay was performed to measure HIV-1 LTR-dependent transcription. Overexpression of TASK-1 significantly inhibited HIV-1 transcription, and this inhibition was dose-dependent (Figure 2a, 2c). Similar results were obtained when cells were co-transfected with TASK-3 and the reporter virus construct (data not shown). Taken together, these results indicate that TASK protein suppresses HIV-1 replication by inhibition of viral transcription.

### TASK channel function is not required for suppression of HIV-1 transcription

**Figure 3:**
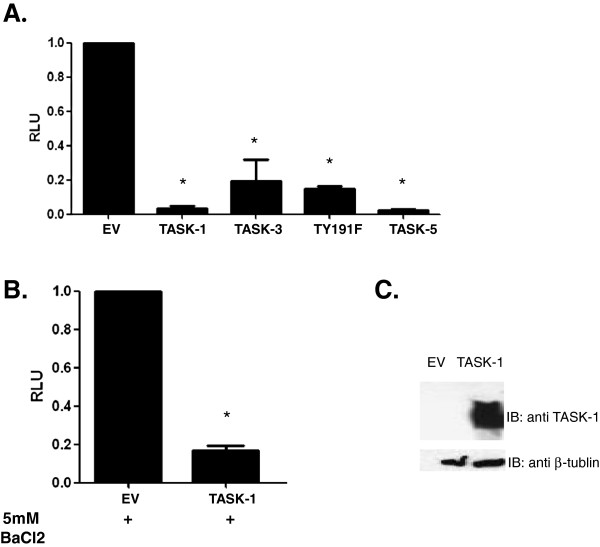
**TASK channel function is not required for suppression of HIV-1 transcription. A**. 293T cells were transfected with pNL4-3Luc and either wild type TASK-1 or non-functional TASK protein expression vectors as described in the text. After 48 hours a luciferase assay was performed to determine the effect of ion channel function on TASK-1’s ability to suppress HIV-1 transcription. Data shown are normalized to empty vector control (EV). **B**. 293T cells were transfected with pNL4-3Luc and wild type TASK-1 in the presence of the ion channel inhibitor 5mM BaCl_2_. After 48 hours a luciferase assay was performed to measure HIV-1 transcription. Data are normalized to the empty vector control (EV). C. Transfected 293T cells were harvested, lysed and equal amounts of cell lysate protein were subjected to 4-12% SDS-PAGE. After transfer to nitrocellulose membranes, TASK and **β**-tubulin proteins were revealed by immunoblotting with polyclonal antibodies followed by chemilumenescence detection. (* P < .001; Statistical analysis was performed on the samples using a student T test).

TASK proteins are potassium channels whose ion channel function contributes significantly to maintaining the resting cellular membrane potential. However several ion channels have phenotypes independent of their ion channel activity. One example, the Ether-à-go-go (EAG) K+ channels regulates intracellular signaling pathways by a mechanism that depends on the position of its voltage sensor [39]. Therefore to examine whether TASK ion channel function was required for suppression of HIV-1 transcription we co-transfected 293T cells with pNL4-3Luc and either a dominant negative TASK-1(Y191F) or TASK-5. TASK-5 is a naturally occurring non-functional TASK protein that is homologous to TASK-1 and TASK-3 [40]. In addition, we added 5mM BaCl_2_ to pharmacologically block TASK function in cells transfected with wild type TASK-1 and a luciferase assay was performed after 48 hours. Our results show that the dominant negative TASK-1 mutant TY191F and the naturally non-functional channel TASK-5 were both able to suppress HIV-1 transcription similar to wildtype TASK proteins (Figure 3a). Furthermore, wild type TASK channels in the presence of 5mM BaCl_2_ were also able to suppress HIV-1 transcription (Figure 3b, 3c). Therefore, TASK ion channel function is not required to suppress HIV-1 transcription. In addition, suppression of HIV-1 transcription appears to be specific to TASK protein family members because an inwardly-rectifying K channel (IRK1), from a different K+ channel family, enhanced rather than suppressed HIV-1 transcription (data not shown).

### Both HIV-1 Vpu and TASK protein decrease HIV-1 transcription

**Figure 4:**
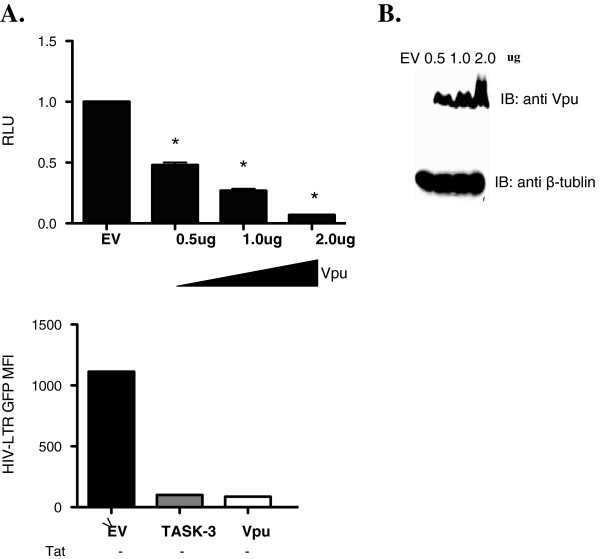
**Vpu suppresses HIV-1 transcription.A**. 293T cells were transfected with pNL4-3 and increasing amounts of Vpu expression plasmid. After 48 hours a luciferase assay was performed to determine the effect of Vpu of HIV-1 transcription. Data are normalized to empty vector control (EV). **B**. Transfected 293T cells were harvested, lysed and equal amounts of cell lysate protein were subjected to 4-12% SDS-PAGE. After transfer to nitrocellulose membranes, VPU and β-tubulin proteins were revealed by immunoblotting with polyclonal antibodies followed by chemilumenescence detection. **C**. 293T cells were transfected with an HIV-1 LTR-GFP construct that does not encode Tat along with TASK-3, Vpu or empty vector. After 48 hours, the cells were harvested and subjected to flow cytometry analysis for GFP expression. Data shown are mean fluorescence intensity of transfected cells. Non-transfected cells were used to set the negative gate. (* P < .001; Student T test was used to determine statistical significance).

As noted above, the first transmembrane domain of TASK-1 (Ttm1) shares amino acid identity with HIV-1 Vpu and can reverse defects in HIV-1 release when added in *trans* to cells transfected with the ∆vpu HIV-1 provirus pNL4-3-Udel [34]. In addition, Vpu has been shown to suppress HIV-1 induced NF-κβ activation [32]. To determine if, like TASK proteins, Vpu could also suppress HIV-1 transcription, 293T cells were cotransfected with pNL4-3Luc and a Vpu expression construct in increasing concentrations. After 48 hours, a luciferase assay was performed to measure viral transcription. Like TASK-1, Vpu significantly suppressed HIV-1 transcription in a dose-dependent manner (Figure 4a, 4b). We also performed a cell viability assay and found no change in cell viability with expression of Vpu (data not shown). Interestingly Vpu suppressed HIV-1 transcription by 90%, a level of suppression even greater than that mediated by TASK-1.

To determine if the HIV-1 Tat protein was involved in suppression of HIV-1 transcription by TASK and Vpu, we co-transfected 293T cells with an HIV-1-LTR-GFP plasmid along with empty vector, TASK-1 or Vpu cDNA in *trans* and measured expression of HIV-1-LTR-dependent GFP by flow cytometry after 48 hours. The HIV-1-LTR-GFP plasmid does not include the *tat* gene and in the absence of Tat there was a low level of endogenous HIV LTR promoter activity. However as previously seen in Figures 2 and 4a both Vpu and TASK significantly suppressed HIV-1 transcription in the absence of Tat (Figure 4c). These results showed that Vpu and TASK-1 suppress HIV-1 transcription in a Tat-independent manner.

### TASK and Vpu proteins preferentially suppress unintegrated HIV-1 DNA

**Figure 5:**
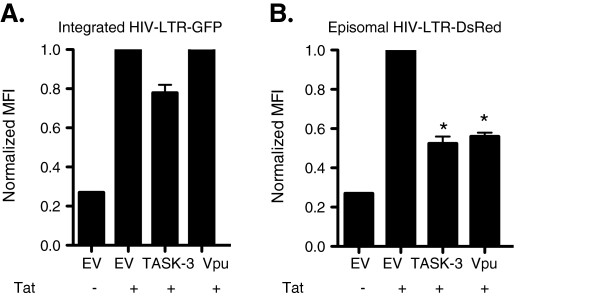
**TASK and Vpu proteins preferentially suppress unintegrated HIV-1 DNA.** JLTRG cells, stable transfectants expressing HIV LTR-GFP construct, were co-transfected with tat, HIV-LTR-DsRed and EV, Vpu or TASK-3GFP. Twenty four hours after transfection the cells were fixed, permeabilized and subjected flow cytometry analysis. The mean fluorescence intensities of the double positive DsRed and GFP populations are indicated in the graphs. (*P < .0005; Student T test was used to determine statistical significance).

We have shown that TASK proteins and Vpu suppress viral transcription in cells transfected with HIV-1 proviral DNA. During these transient transfection assays, the HIV-1 provirus remains unintegrated. Therefore we sought to examine the effect of TASK and Vpu proteins on HIV-1 transcription from integrated and unintegrated viral genomes. For this purpose we determined the effect of TASK and Vpu expression on HIV-1 transcription in cells that had contained an integrated and an unintegrated viral genome. We used the JLTRG cell line which is derived from the Jurkat cell line and has been stably transfected with an HIV LTR-GFP construct. The JLTRG cells were transfected with a Tat expression plasmid along with HIV-LTR-DsRed, TASK or Vpu expression vectors or empty vector control. In the absence of HIV-1 infection or HIV-1 Tat expression the cells exhibit no GFP expression. Twenty four hours after transfection we used flow cytometry to assesses the expression of GFP and DsRed as a measure of LTR activity and plotted the respective MFI. As previously observed, both TASK and Vpu proteins significantly suppressed transcription of the unintegrated HIV-LTR-DsRed (Figure 5b). Interestingly, both TASK and Vpu had only limited affect on transcription from the integrated HIV LTR-GFP construct (Figure 5a). Taken together these results indicate that TASK proteins and Vpu preferentially suppress transcription of uDNA in cells containing both integrated and uDNA.

### TASK-1 and Vpu suppress HIV transcription in cells infected with Integrase defective virus

**Figure 6:**
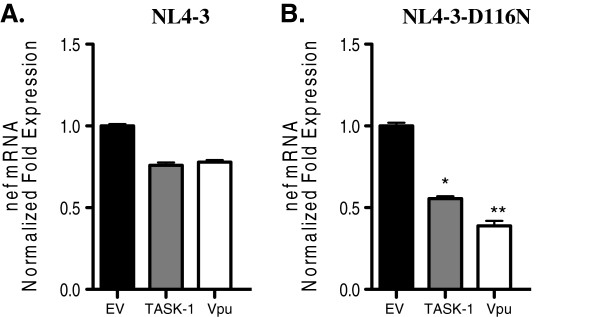
**TASK-1 and Vpu preferentially suppress transcription of cells infected with Integrase defective HIV-1.** Jurkat cells were transfected with empty vector (EV), TASK-1 or Vpu expression plasmids and then infected with wildtype NL4-3-CFP (**A**) or NL4-3D116N-YFP (**B**). Twenty four hours after infection the cells were lysed and total RNA was isolated and used as template for real-time PCR to measure nef mRNA. Data shown are normalized to EV control. (* P = .0002; **P < .0001; Student T test was used to determine statistical significance).

Unintegrated copies of HIV-1 DNA are enriched in cells infected with integrase-defective viruses. We took advantage of this phenomenon to confirm that Vpu and TASK-1 proteins suppress transcription of uDNA. We transfected Jurkat cells with either TASK-1 or Vpu and then infected the cells with wildtype NL4-3-CFP or integrase-defective NL4-3-D116N-YFP HIV-1. Thirty hours after infection, RNA was isolated and used as template for real-time PCR analysis of nef mRNA. As seen in Figure 6b, TASK and Vpu proteins significantly suppressed nef transcription in cells infected with integrase defective HIV-1 and minimally suppressed transcription in cells infected with wildtype HIV-1 (Figure 6a). These data confirmed our earlier results and strongly indicated that TASK and Vpu preferentially suppress transcription of uDNA.

### Vpu and TASK-1 suppress unintegrated HIV-1 DNA in an NFκβ-dependent manner

**Figure 7:**
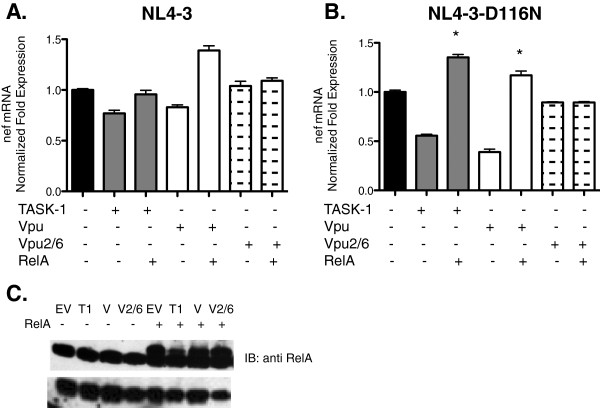
**Suppression of HIV-1 transcription by Vpu and TASK-1 is abrogated by overexpression of RelA.** Jurkat cells were transfected with EV, TASK-1 or Vpu or EV + RelA, TASK-1 + RelA or Vpu + RelA and then infected with wildtype NL4-3CFP (**A**) or NL4-3-D116N-YFP (**B**). Twenty four hours after infection the cells were lysed and total RNA was isolated and used as template for real-time PCR to measure nef mRNA. Data shown are normalized to empty vector control (**C**). Transfected Jurkat cells were harvested, lysed and equal amounts of lysate protein were subjected to 4-12% SDS-PAGE. After transfer to nitrocellulose membranes, Vpu, RelA and β-tubulin proteins were revealed by immunoblotting with polyclonal antibodies and visualized by chemilumenescence detection. (* P < .0001; Student T test was used to determine statistical significance).

In a previous study, Bour et al. demonstrated that Vpu interferes with NF-κβ activation by preventing degradation of Iκβ [32]. We explored this phenomenon as a possible mechanism by which Vpu and TASK suppress HIV-1 transcription. We transfected Jurkat cells with empty vector, vectors expressing TASK-1, Vpu, or phosphorylation defective Vpu2/6 along with empty vector or RelA(NF-κβ) in trans. Vpu2/6 does not cause degradation of Iκβ and subsequent NF-κβ activation. The cells were then infected with either wildtype or integrase defective HIV-1. Thirty hours after infection we isolated RNA for real-time PCR analysis of nef mRNA. Vpu and TASK minimally suppressed transcription of wild type HIV-1 (Figure 7a). However, as previously observed, both proteins suppressed transcription of integrase defective HIV-1 (Figure 7b). Interestingly, overexpression of RelA reversed the suppression of transcription by both Vpu and TASK proteins in cells infected with integrase-defective HIV-1 (Figure 7a, 7b, 7c). These results show that both Vpu and TASK suppress HIV-1 transcription through an NFκβ-dependent mechanism. Our findings are consistent with the findings of Bour et. al. that Vpu inhibits βTrCP-dependent degradation of Iκβ since phosphorylation defective Vpu2/6, which does not interfere with Iκβ degradation and subsequent NF-κβ activation, did not suppress HIV-1 transcription. Furthermore overexpression of RelA in the presence of Vpu2/6 did not increase nef mRNA as was observed for overexpression of RelA in wildtype Vpu samples.

## Discussion

Hsu et al. showed that a cellular K+ channel TASK-1 is homologous to HIV-1 Vpu and that the first transmembrane domain of TASK-1 was functionally interchangeable with Vpu [34]. They also observed that TASK-1 was able to inhibit HIV-1 release and appeared to function as an HIV-1 restriction factor. We wanted to follow up on this observation and examine potential mechanisms by which TASK proteins block HIV-1 replication. Using a combination of transfection experiments and infections by wildtype and integrase-defective virus, we obtained data indicating that Vpu and TASK proteins suppress transcription of HIV-1 genes. This effect was preferentially exerted on uDNA. The mechanism by which TASK and Vpu differentially suppress transcription from uDNA is not clear. We showed that the mechanism likely involves NF-κβ and it is not clear why this factor would have a differential effect on integrated versus uDNA. It is possible that other critical proteins required for transcription from uDNA but not integrated genomes are either sequestered or degraded in the presence of Vpu or TASK. Integrated HIV DNA as part of the cellular chromatin structure may require a different set of cellular factors for transcription compared to uDNA.

HIV-1 Vpu is a late gene product expressed after HIV-1 integration when sufficient levels of Rev is present for the production of unspliced and singly spliced genes [41]. In addition to antagonizing the host protein tetherin, degrading CD4 and enhancing virus release, Vpu decreases both TNF-α and HIV-1-mediated activation of NF-κβ by inhibiting βTrCP-dependent degradation of Iκβ [32]. Other viral proteins such as Vpr, Tat, and gp120 have also been shown to directly or indirectly affect NF-κβ regulation [42-46]. Prior to HIV-1 integration, uDNA produce early gene products: nef, rev and tat. After integration of HIV-1 proviral DNA, when sufficient Rev levels are present, Vpu mRNA is translated and the protein accumulates in infected cells. HIV-1 infection causes constitutive activation of NF-κβ [47-49], a host transcription activator that resides in the cytoplasm complexed to Iκβ. βTrCP-mediated degradation of Iκβ frees NFκβ to migrate into the nucleus where it can bind a variety of promoters including the HIV-1 LTR. Using immunoprecipitation studies, Hsu et. al demonstrated an association between Vpu, TASK-1 and βTrCP. They also showed that TASK-1 protein is degraded after infection in primary CD4^+^ T lymphocytes [34]. These findings the led authors to speculate that Vpu may mediate the degradation of TASK-1 through the βTrCP ubiquitin ligase pathway similar to Vpu-mediated degradation of CD4 and Iκβ [32,34]. Our findings with both functional and nonfunctional TASK proteins along with the previously described interaction between TASK-1 and βTrCP indicate that TASK-1 and Vpu may share a common mechanism of suppressing HIV-1 transcription: decreased NF-κβ activation by inhibiting βTrCP-dependent Iκβ degradation.

Bour et al. showed that overexpression of sub-genomic constructs expressing all HIV-1 proteins except Gag and Pol led to a moderate 3-fold activation of NF-κβ in the presence of Vpu and a 14-fold enhancement in the absence of Vpu [32]. Similar results were obtained when full-length HIV-1 constructs were used. Thus Vpu negatively modulates of NF-κβ activation and this would lead to lower levels of HIV-1 transcription in some cell types. This data is consistent with results presented here where we demonstrate that suppression of HIV-1 transcription by Vpu and TASK proteins can be abrogated by overexpression of RelA. Interestingly, our results show that Vpu and TASK proteins exert their negative effect on HIV-1 transcription preferentially on uDNA. Our findings are not in conflict with those of Bour et al. since they used largely transfection-based studies to examine the effect of Vpu on NF-κβ activation. In such studies, proviral genomes remain unintegrated.

We also demonstrated that homologous TASK proteins profoundly suppressed HIV-1 transcription. TASK-1 and TASK-3 proteins are constitutively expressed in primary CD4^+^ T cells and contribute 40% of the K^+^ efflux needed to mediate Ca^2+^-dependent T cell effector function, namely T cell proliferation and the production of IFNγ and IL-2 [50]. IFNγ induces TNFα production leading to NF-κβ activation and subsequently HIV-1 LTR mediated transcription. T cell effector function is a Ca^2+^ dependent immune response that also leads to the destruction of virally infected cells and the priming of neighboring T cells [50]. TASK-mediated enhancement of Ca^2+^-dependent transcription of cytokines after HIV-1 infection may be a component of the host cell response to restrict HIV-1 replication. Since TASK proteins can suppress HIV-1 transcription and they play a role in generating critical cytokines needed to combat the virus, TASK proteins can be viewed as HIV-1 host restriction factors. This is consistent with the observation that after HIV-1 infection, Vpu leads to the degradation of TASK proteins [34]. However, modulation of transcription from uDNA may be essential to efficient replication as this function is present in Vpu and therefore maintained by the virus.

Our data and model are not in conflict with the earlier studies of Wu and others showing efficient expression of early genes from episomal HIV DNA [20,24,30,41,51-54]. In our model, TASK, as a constitutively expressed cellular protein, would suppress expression of early genes from episomal DNA as part of the cellular response to the virus. TASK may represent the natural silencing mechanism and Vpu, which is able to cause degradation of TASK, may represent the virus’ mechanism for suppressing the cellular response. It is extremely intriguing, that the virus protein that is able to destroy the restricting protein (TASK) has the same activity itself. Thus, initially Vpu’s role may be to suppress TASK and then later it suppresses transcription from uDNA after sufficient early gene products have formed to drive expression from integrated DNA.

## Conclusion

Our findings have important implications given the increased use of integrase inhibitors in antiretroviral therapy. In the presence of integrase inhibitors uDNA accumulates because more substrates are available for the non-homologous end joining pathways when integration is blocked [55,56]. Although uDNA templates are transcriptionally active and can be suppressed by TASK protein, the presence of uDNA prior to integration has been associated with activation and productive infection in resting T-cells via expression of Tat and Nef from uDNA. When HIV-1 integration is blocked CD4, CCR5 and CXCR4 are down regulated by Nef, leading to a restriction of superinfection events [57-60]. In addition, HLA molecules recognized by helper [61] and cytotoxic T-cells are down regulated by Nef while HLA molecules that are recognized by natural killer cells are not affected by Nef [62-65]. Therefore early production of Nef from uDNA may be important for immune evasion by HIV-1 [61]. In non-dividing cells with slower viral replication kinetics, such as macrophages, uDNA is biologically active and very stable. In these cells there is persistent transcription, primarily of early genes, of the uDNA. In infections with integrase defective lentiviral vectors in animal models, reservoirs of uDNA are relatively long lived [66,67].

The regulation of uDNA is important since replication of integrase-defective viruses can be rescued by superinfection with wild type virus [26,68]. Vpr promotes transcription from the uDNA, and after integration when Rev protein is present, transcripts from uDNA can be exported from the nucleus and translated. Therefore proteins encoded by uDNA can participate in virion assembly. Both RNA and protein products from uDNA can be packaged into virions, providing an opportunity for recombination and second round infections [68]. This phenomenon could contribute meaningfully to the genetic diversity of HIV-1.

## Materials and methods

### Reagents, plasmids, and antibodies

TY191F [69], rodent TASK-1, rodent TASK-3 and IRK-1 expression plasmids were kind gifts from Dr. Douglas Bayliss (University of Virginia Charlottesville, VA). Human TASK-1, human TASK-3 and RelA-myc expression vectors were obtained from Origene (Rockville, MD). The Vpu-eGFP expression vector was a kind gift from Dr. Vasundhara Varthakavi (National Institutes of Health Bethesda, MD). Expression plasmids for codon-optimized Vpu (pcDNA-Vphu), phosphorylation defective mutant pcDNA-Vphu2/6 and a polyclonal antibody to Vpu were generous gifts from Klaus Strebel (National Institutes of Health, Bethesda, MD) and have been described elsewhere [32]. A monoclonal antibody against HIV-1 Gag (GagM1) was generated in our laboratory. Commercially available antibodies against HIV-1 Gag KC57-RD1 (Beckman Coulter), TASK-1 (Chemicon) and β-tubulin (Santa Cruz Biotechnology) were used for immunoblotting experiments. Secondary antibodies (horseradish peroxidase-conjugated goat anti-rabbit (HRP-GAM), goat anti-mouse H+L (GAMH+L) and Fcγ specific (GAMF_c_) were obtained from Jackson ImmunoResearch Laboratories. Cell viability was measured using the Invitrogen fluorescence based LIVE/DEAD cell assay kit.

### Viruses

HIV-1 was prepared in 293T cells by transfection of plasmid DNA (pNL4-3-CFP and pNL4-3 D116N-YFP (kind gifts from Dr. David Levi) [26], and pNL4-3, pNL4-3-Udel (kind gift from Dr. Klaus Strebel)) using Lipofectamine (Invitrogen) per the manufacturer's instructions. The supernatants containing virus particles were harvested after 48hr of culture, filtered through a 0.2 micron filter and pelleted through a 20% sucrose cushion (100,000×g, 1hr). All virus preparations were treated with DNase I to remove plasmid DNA.

### Cells

The Jurkat T cell line was obtained from the American Type Culture Collection (ATCC) and cultured in complete RPMI (cRPMI) (RPMI, 10 mM HEPES, 2 mM L-glutamine, 10% fetal bovine serum (FBS). The JLTRG cell line was obtained from Dr. Olaf Kutsch through the AIDS Research and Reference Reagent Program, Division of AIDS, NIAID, NIH: JLTRG (Cat #11587). HEK 293T cell line was obtained from the ATCC and cultured in complete Dulbecco modified Eagle medium (cDMEM) DMEM, 10 mM HEPES, 2 mM L-glutamine, 10% fetal bovine serum (FBS). Cell viability was assessed by trypan blue exclusion.

### Infections and transfections

HEK 293T cells were plated in 60-cm dishes (5 × 10^5^ cells/2ml) and co-transfected with pNL4-3-Luc and TASK or Vpu cDNA. After washing and 48 hours of culture as above, the cells were lysed and lysates were analyzed for luciferase activity with the Promega luciferase assay kit as per manufacturer’s instructions. Jurkat cells were transfected with Vpu-eGFP or TASK-GFP DNA using the human T cell Nucleofector system (Amaxa, Gaithersburg, MD) per the manufacturer's instructions. After 6 hours cells were infected by spinoculation with 50ng of either wildtype NL4-3-CFP or integrase defective NL4-3D116N-YFP virus. Infection and transfection efficiencies were determined by either western blot analysis or flow cytometry. Briefly, for flow cytometry, cells were fixed with 2% paraformaldehyde, permeabilized using 0.1% saponin, and then stained with the mouse KC57-RD1 (anti-Gag) or an isotype-matched control antibody. Stained cells were analyzed on a Beckman FACS Calibur flow cytometer equipped with Cell Quest software. Viral infection and transfection rates were expressed as percentages of cells staining positively with the anti-p24 antibody or expressing CFP, YFP or GFP as appropriate.

### Virus titer determinations

Two days after transfection with virus DNA constructs, HIV-1 Gag was measured in the cell culture supernatants and cell lysates using a capture ELISA.

### Sodium dodecyl sulfate-polyacrylamide gel electrophoresis and immunoblotting

Cell lysates were prepared by adding RIPA lysis buffer containing protease inhibitor cocktail (Roche) to cell pellets followed by incubation for 30 min on ice. Protein concentrations of cell lysates were determined using a bicinchoninic acid protein assay kit (Pierce-Rockford, IL). Equal amounts of protein were taken up in gel loading buffer and the samples were heated at 70°C for 10 min before loading onto a 4 - 12% sodium dodecyl sulfate-polyacrylamide gel electrophoresis gradient gels (NuPAGE Novex Bis-Tris gels; Invitrogen). Proteins were transferred onto a Hybond-P polyvinylidene difluoride membrane (Amersham Biosciences, NJ) following manufacturers protocol. The membranes were blocked in 5% powdered milk in PBS, 0.1% Tween 20 and then probed with TASK-1 (1:1000), Vpu (1:000) or NF-κβ (1:500 dilution) for 60 min at 25°C . The membranes were then incubated for 60 min with horseradish peroxidase-conjugated, anti-mouse (1:10,000) or anti-rabbit (1:10,000) immunoglobulin G secondary antibodies (Promega, San Luis Obispo, CA) and developed using Super Signal chemiluminescence (Pierce). Bands were visualized on Denville Scientific film.

### Real-time PCR

Total RNA was isolated using a Qiagen total RNA purification kit as per manufacturer’s instructions. RNA was treated with Qiagen’s DNase I and reverse-transcribed using the Invitrogen Superscript cDNA synthesis kit. The cDNA was treated with RNase H and then subjected to real time PCR with HIV-1 gene-specific primers. Real-time PCR conditions and primers to detect HIV-1 Nef-2 mRNA are described in detail elsewhere [70].

### Statistical analysis

All experiments were performed three to six times, and representative experiments are shown. The Student *t* test was used to determine the statistical significance of the results.

## Abbreviations

TASK: Twik-related acid sensitive K channel; Vpu: Viral protein U; uDNA: unintegrated HIV DNA; IRK: inward rectifying K+ channel; NF-κβ: nuclear Factor kappa-light-chain-enhancer of activated B cells; Iκβ: I kappa beta; RelA: NF-κβ(p65); βTrCP: beta-transducin repeat containing protein.

## Competing interests

The authors of this manuscript have not in the past five years received reimbursements, fees, funding, or salary from an organization that may in any way gain or lose financially from the publication of this manuscript, either now or in the future. They do not hold any stocks or shares in an organization that may in any way gain or lose financially from the publication of this manuscript, either now or in the future. They do not hold or are not currently applying for any patents relating to the content of the manuscript. They have not received reimbursements, fees, funding, or salary from an organization that holds or has applied for patents relating to the content of the manuscript. The authors of this manuscript do not have any other financial competing interests. Lastly, the authors of this manuscript do not have any non-financial competing interests (political, personal, religious, academic, ideological, intellectual, commercial or any other) to declare in relation to this manuscript.

## Authors’ contributions

NE carried out the transfections, infections, biochemical assays, immunoassays and drafted the manuscript. JH conceived the study, participated in its design and coordination and edited the manuscript. Both authors read and approved the final manuscript.
